# Phillygenin Inhibits *Helicobacter pylori* by Preventing Biofilm Formation and Inducing ATP Leakage

**DOI:** 10.3389/fmicb.2022.863624

**Published:** 2022-04-28

**Authors:** Ru-Jia Li, Chun Qin, Gan-Rong Huang, Li-Juan Liao, Xiao-Qiang Mo, Yan-Qiang Huang

**Affiliations:** Research Center for the Prevention and Treatment of Drug Resistant Microbial Infecting, Youjiang Medical University for Nationalities, Baise, China

**Keywords:** *Helicobacter pylori*, *Forsythia suspensa*, phillygenin, bacteriostasis, mechanism

## Abstract

With the widespread use and abuse of antibiotics, *Helicobacter pylori* (*H. pylori*) has become seriously drug resistant. The development of new antibiotics is an important way to solve *H. pylori*'s drug resistance. Screening antibacterial ingredients from natural products is a convenient way to develop new antibiotics. Phillygenin, an effective antibacterial component, was selected from the natural product, forsythia, in this study. Its minimal inhibitory concentration (MIC) for 18 *H. pylori* strains was 16–32 μg/ml. The minimum bactericidal concentration (MBC) of *H. pylori* G27 was 128 μg/ml; the higher the drug concentration and the longer the time, the better the sterilization effect. It was non-toxic to gastric epithelial cell (GES)-1 and BGC823 cells at the concentration of 100 μg/ml. It presented a better antibacterial effect on *H. pylori* in an acidic environment, and after 24 days of induction on *H. pylori* with 1/4 MIC of phillygenin, no change was found in the MIC of *H. pylori*. In the mechanism of action, phillygenin could cause ATP leakage and inhibit the biofilm formation; the latter was associated with the regulation of *spoT* and *Hp1174* genes. In addition, phillygenin could regulate the genes of *Nhac, caggamma, MATE, MdoB, flagellinA*, and *lptB*, leading to the weakening of *H. pylori*'s acid resistance and virulence, the diminishing of *H. pylori*'s capacity for drug efflux, *H. pylori*'s DNA methylation, the initiation of human immune response, and the ATP leakage of *H. pylori*, thus accelerating the death of *H. pylori*. In conclusion, phillygenin was a main ingredient inhibiting *H. pylori* in *Forsythia suspensa*, with a good antibacterial activity, high safety, strong specificity, better antibacterial effect under acidic conditions, and low risk of resistance development by *H. pylori*. Its mechanism of action was mainly associated with inhibiting the biofilm formation and resulting in ATP leakage. In addition, phillygenin was shown to be able to reduce the acid resistance and virulence of *H. pylori*.

## Introduction

*Helicobacter pylori (H. pylori)* is an important cause of multiple extra gastrointestinal diseases, chronic gastritis, peptic ulcer, and gastric cancer (Amnon et al., 2010; Plummer et al., 2016; Sultan et al., 2016). At present, the treatment of Western medicine to eradicate *H. pylori* mainly includes the standard triple therapy, the non-tantalum four-link schemes, the bismuth-based quadruple therapy, etc. (Nagy et al., 2016), the increasingly higher resistance rate, and increasingly lower eradication rate of *H. pylori*, which are attributed to the long-term use, and abuse of antibiotics poses a serious threat to the health and safety of the public (Hooi et al., 2017). However, the search for new therapeutic drugs is the fundamental solution to the current problem, and screening effective ingredients from the bacteriostatic natural products serves as a convenient way to develop new drugs. *Forsythia suspensa* (*F. suspensa*), a kind of natural plant from the *Forsythia* of *Oleaceae*, is a precious traditional Chinese medicinal material (Yang et al., 2019) that contains active ingredients such as the coumarin, phillyrin, and rutin, with antibacterial, antitumor, and anti-inflammatory properties and the ability to enhance immunity (Seung et al., 2009; Yuan et al., 2015; Bae et al., 2020). *Forsythia suspensa* has various components with complex mechanisms of action; it is difficult to determine on which microorganism it has a better inhibitory effect and to identify an effective active ingredient from it and explore this ingredient's mechanism of action. Dong et al. (2016) reported that *F. suspensa* had a certain inhibitory effects on *H. pylori* with no discussing its functions, ingredients, and mechanism. In this study, phillygenin was screened from *F. suspensa* as the main antibacterial ingredient for *H. pylori* and evaluated as having a good druggability in terms of its toxicity, antibacterial spectrum, acid resistance, and drug resistance, besides the exploration of phillygenin's mechanism through experiments on inhibiting biofilm formation, ATP leakage detection, the transcriptome detection and verification, etc.

## Materials and Methods

### Recovery and Cultivation of Bacterial Strains

*Helicobacter pylori* strains containing the preserving liquid were removed from the refrigerator where they were stored at −80°C (standard strains 26695, NSH57, MSD132, and G27 were presented by professor Hongkai Bi from Nanjing Medical University; the clinical strains HPBS001-HPBS016 were isolated by our laboratory), Information on other strains are provided in Supplementary Table 1. *Helicobacter pylori* strains were cultured on the Columbia blood agar plate (OXOID, UK) or in the brain heart infusion (BHI, OXOID, UK) broth medium containing 10% serum (Pingrui, China). Bacterial species from non-*H. pylori* group were used as an experimental control and were cultured on nutrient agar (NA) plates or Luria-Bertani (LB) plates.

### Detection of MIC

The active ingredients of *F. suspensa* purchased from Chengdu Herbpurify Co., Ltd. (CAS: 487-39-8, etc.) were dissolved into 4 mg/ml using absolute ethanol. The first well of a 96-well plate with bacteria was added with 173.6 μl of culture medium and then with 6.4 μl of antibacterial drugs, and every well from the first to the eighth was all diluted in proportion (the drug concentrations of the first to eighth wells were 128, 64, 32, 16, 8, 4, 2, and 1 μg/ml) with negative wells (sterile, only with medium, and drugs) and positive wells (no drugs, only with medium, and bacteria) as controls. Bacteria at the logarithmic growth phase were taken from the solid plate and made into bacterial suspensions with the corresponding mediums: the concentration of *H. pylori* was adjusted to 0.3 [1.0 × 10^8^ colony forming unit (CFU)/ml] and diluted 10 times to 1.0 × 10^7^ CFU/ml. The concentration of other bacteria at OD600 was adjusted to 0.3 (1 × 10^8^ CFU/ml) and diluted 100 times to 1 × 10^6^ CFU/ml. The concentration of fungi was adjusted to 0.5 (5 × 10^6^ CFU/ml) and diluted 1,000 times to 5 × 10^3^ CFU/ml. Notably, 10 μl was taken and added from the first to eighth well (the concentration of bacterium per well was about 1.0 × 10^6^ CFU/ml) and the wells with bacteria were cultivated for 24–72 h to judge the results (Huang et al., 2019).

### Detection of MBC

Phillygenin was dissolved to 4 mg/ml by absolute ethanol. The first well of the 96-well plate with bacteria was added with 173.6 μl culture medium (pH 3.0, pH 4.5, pH 6.0, and pH 7.0) with 90 μl culture medium added to the other wells. Thereafter, 6.4 ml of phillygenin was added to the first well, with every well from the first to the fifth well diluted in proportion (the drug concentrations were 128, 64, 32, and 16, 8 μg/ml) and phosphate-buffered saline (PBS) (sangon, China) used as a positive control. The *H. pylori* G27 strains at the logarithmic growth phase on the solid medium were employed to make a bacterial suspension with a BHI medium; the concentration of the bacteria liquid was adjusted to 1 × 10^8^ CFU/ml (OD600 was 0.3), diluted by ten times, and kept in reserve. The first to fifth wells were added with 10 μl of the bacteria liquid (the concentration of bacteria liquid per well was about 1 × 10^6^ CFU/ml) and cultured in a three-gas incubator. The bacteria liquid after the drug action for a certain period of time (such as 2 h) was diluted (100 times, 1,000 times, etc.), coated on a Columbia agar plate, and cultured in a three-gas incubator for 4–5 days. The number of bacteria growing on the agar plate was calculated, with the drug concentration at which the number of bacteria was suppressed by 99.9% as the MBC.

### Drug Resistance Detection of Phillygenin

*Helicobacter pylori* G27 strain was used to detect the drug resistance of phillygenin. First, the MICs for *metronidazole* and phillygenin were 2 and 16 μg/ml, respectively. The induction on the strains was performed with one-fourth MIC concentration of *metronidazole* and phillygenin, with the detection performed every 3 days during a total of 24 days of induction. The induced concentrations were adjusted with the change of MICs. For example, the induced concentration was adjusted to 4 μg/ml when the MIC of metronidazole was 16 μg/ml.

### Cytotoxicity Test of Phillygenin

The cell suspensions of gastric epithelial cell (GES)-1 and BGC823 (KeyGEN BioTECH, Nanjing, China) were prepared with their concentrations adjusted to 1 × 10^5^. The suspensions were then inoculated into a 96-well plate, 100 μl per well. Three repeats of the same sample were performed and cultured in the incubator at 37°C for 24 h. Moreover, 10 μl of phillygenin (the working concentrations were 300, 200, 100, 50, and 0 μg/ml) was added to each well and inoculated at 37°C for 24 h. Furthermore, 10 μl of CCK8 (Beyotime, China) was added per well, tapped, and mixed well and then incubated for 4 h. The absorbance at 450 nm was measured, and the survival rate was calculated according to the formula: cell survival rate = [(As-Ab)]/[(Ac-Ab)] × 100%. As refers to the wells that contain the culture medium of cells, drugs, and CCK-8. Ac refers to the wells that contain the culture medium of cells and CCK-8 with no drug. Ab refers to the wells that contain the culture medium of cells and CCK-8, with no cell and drug. A survival curve based on the survival rate was established.

### Animal Toxicity Test of Phillygenin

Specific pathogen-free (SPF) C57BL/6 mice aged 6–8 weeks were purchased from Changsha Tianqin Biological Co., Ltd., the number of SPF animal license: SYXK Gui 2017-0004, and animal experiment Ethics Number: No. 2019112501. The evaluation of drug efficacy for animal *in vivo* as follows was performed in accordance with the same experiment ethics. The mice were raised in an SPF environment and randomly divided into the medicine administration group and negative control group with 10 in each group. Ten times therapeutic dose of phillygenin was administered to the treatment group for 3 consecutive days, once a day; the negative control group was given the PBS buffer solution with the same times of administration and dosage as the administration group. The mice were weighed a day before the administration and weighed after the administration for 7 days. On the third day after the drug withdrawal, the mice in the infection group were weighed, and their average weight was calculated. The blood from their eyeballs was collected. Thereafter, the mice were sacrificed by cervical dislocation, their stomachs, kidneys, livers, and spleens were made into pathological sections, stained with H & E.

### Detection of the Phillygenin Inhibitory Effect *in vivo*

Phillygenin, omeprazole (Sigma-Aldrich, Germany), amoxicillin (Sigma-Aldrich, Germany), and clarithromycin (Sigma-Aldrich, Germany) were dissolved and diluted to 10 mg/ml. The models of SPF C57BL/6 mice aged 6 weeks were established (HPBS001). The mice were divided into four groups, namely, the omeprazole + amoxicillin + clarithromycin group (the triple-therapy group), the omeprazole + phillygenin group (28 mg/kg), the omeprazole + phillygenin group (7 mg/kg), and the PBS group, each with 10 mice. In addition, 10 mice that were not infected with *H. pylori* were treated as the negative control. The treatment group was given an intragastric administration. The groups that contained omeprazole were administered omeprazole first and then other drugs 30 min later. After the administration, the mice were made to fast and deprived of water for 4 h. The dosage was 138.2 mg/kg of omeprazole, 28.5 mg/kg of amoxicillin, and 14.3 mg/kg of clarithromycin, once a day for 3 consecutive days; the control group was given the PBS buffer solution with the same volume and times of administration as above. Two days after the drug withdrawal, the blood was collected from the eyeballs of the mice, which were then sacrificed by cervical dislocation with their stomach tissues taken and broken to acquire *H. pylori* that was then isolated, cultured, and identified with the amount of colonization calculated. A part of the stomach tissues was made into paraffin sections with H&E staining, TUNEL immunohistochemistry, and fluorescence immunoassay performed thereon.

### Inhibition Experiment of Phillygenin on *H. pylori's* Biofilms

The OD of the *H. pylori* G27 bacterial suspension was adjusted to 0.1 and inoculated into a 96-well plate under microaerobic conditions at 37°C for 3 days to form *H. pylori* biofilms (Hathroubi et al., 2020), which were then added with phillygenin (concentrations were 128, 64, 32, and 16 μg/ml). The antibiofilm effect of phillygenin was evaluated through crystal violet (Macklin, China) staining and detection using the Alamar blue assay (Solarbio, China). Biofilms were stained with LIVE/DEAD BacLight Bacterial Viability Kit (Thermo Fisher, America) in the dark for 15 min and then visualized using the confocal laser scanning microscopy. The wavelength of the SYTO 9-stained live cells that had been activated was 411 nm, while that of the PI-stained dead cells was 568 nm. The protein content of these biofilms was determined by the BCA Protein Quantification Kit (Beyotime, China).

### Inhibition Experiment of Phillygenin on ATP

*Helicobacter pylori* G27 was cultured to the logarithmic phase, and the concentration of the bacteria solution was adjusted to 1 × 10^7^ CFU/ml. Meanwhile, three gradients of the working concentration of phillygenin were set, which were 32, 64, and 128 μg/ml, respectively. Polymyxin B (Macklin, China) was used for the positive control and PBS for the negative control. They were cultured for 2 h and centrifuged to acquire the supernatant and bacteria. Adenosine triphosphate (ATP) was detected by ATP Assay Kit (Beyotime, China) through the multifunctional enzyme microplate reader (BioTek, USA).

### Transcriptomics Detection

In the exploration of the semi-inhibition curve of phillygenin, the concentration of the bacterial suspension was adjusted to 1 × 10^8^ CFU/ml, with drug action lasting (16, 32, 64, and 128 μg/ml) for 0, 2, and 8 h. Thereafter, the OD value at 600 nm (HITACHI, Japan) was measured with the semi-inhibitory drug concentration confirmed when the OD value remained unchanged. The bacteria were extracted for a transcriptome sequencing, which was completed by Nanjing Fengzi Bio-pharm Technology using the second-generation Illumina high-throughput sequencing platform and PE150 sequencing strategy. As a result, a total of 80,448,750 raw read pairs were measured with 79,746,845 clean read pairs obtained after quality control. Bowtie2 and Rockhopper were employed to carry out the comparison and sliced transcript analysis. Thereafter, all genes were quantitatively analyzed, and a total of 1,214 differential genes were identified. A functional enrichment analysis was performed on these differential genes to explore their features.

### Reverse Transcription and RT-qPCR

*Helicobacter pylori* G27 was cultured to the logarithmic phase with the concentration of the bacterial solution adjusted to 1 × 10^8^ CFU/ml. The bacterial solution was added with phillygenin for a certain time based on the semi-inhibitory growth curve and centrifuged to obtain the precipitate. Zero hour was labeled as F_1 group, 2 h as F_2 group, and 8 h as F_3 group. Three biological repeats were performed in each group, labeled as A, B, and C. The TRIZOL reagent (Takara, China) was used to extract RNA, the transcription of which was reversed into cDNA through a reverse transcription kit (MonPure, China). RT-qPCR was performed applying the LightCycler according to the RT-PCR kit (MonPure, China); the sample was subjected to 95° predenaturation for 30 s, 95° denaturation for 10 s, and 60° annealing and extension for 30 s, about 40 cycles. The primers are displayed in Supplementary Table 2. The 16S rRNA amplicon (Thermo Fisher, America) was used as an internal control for data standardization, and the remaining primers were purchased from Sangon Biotech (Shanghai). The change at the transcriptional level was determined by applying the relative quantification method (2^−ΔΔCT^). The dissociation curve was analyzed to verify the homogeneity of the products.

### Statistical Methods

All data were expressed as mean ± standard deviation (SD). Data analyzed by one-way analysis of variance were performed using SPSS 25.0 and *p* < 0.05 considered to be statistically significant.

## Results

### Antibacterial Effect of Phillygenin *in vitro*

**Figure 1:**
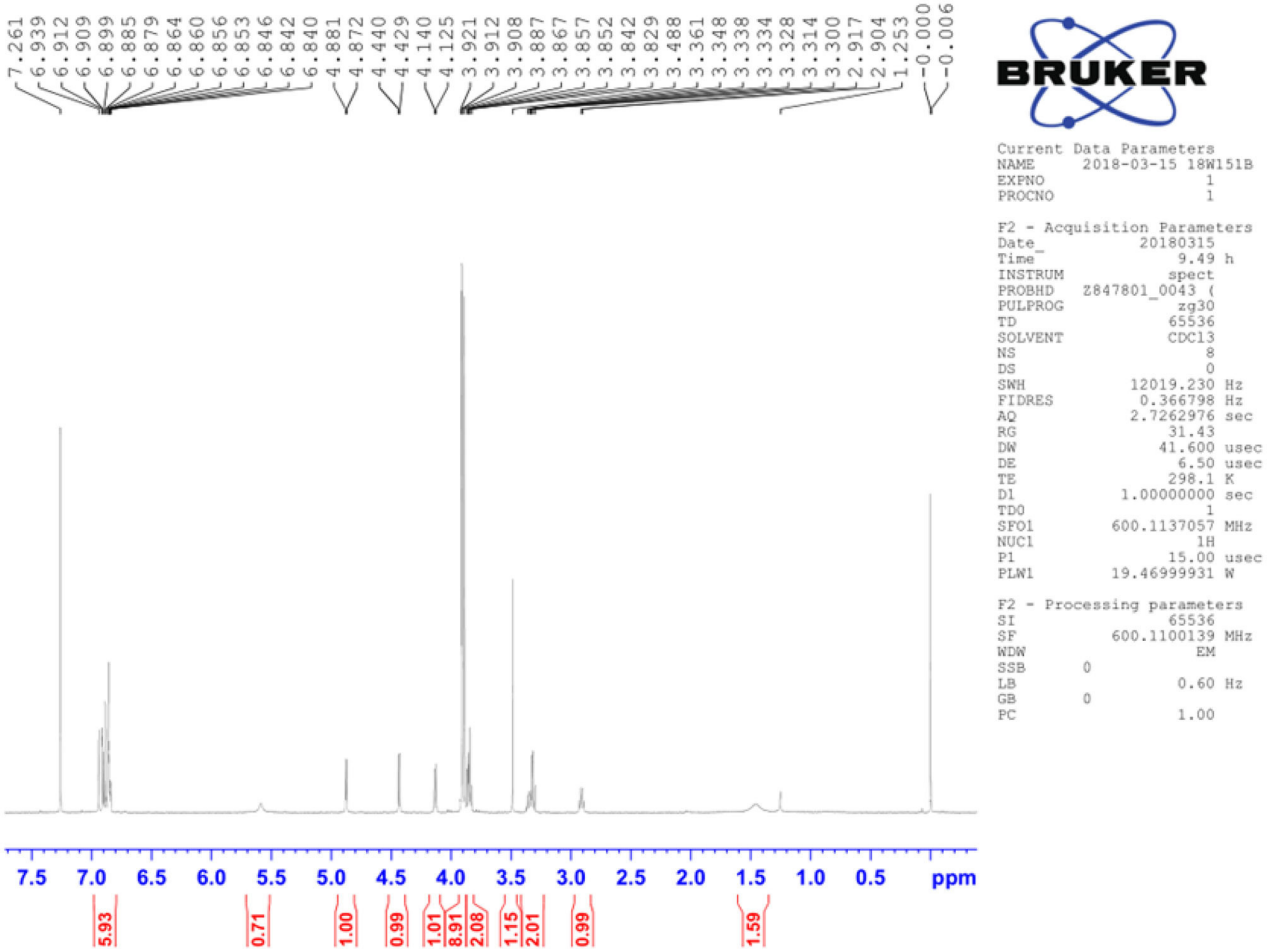
Liquid chromatogram of phillygenin.

**Figure 2:**
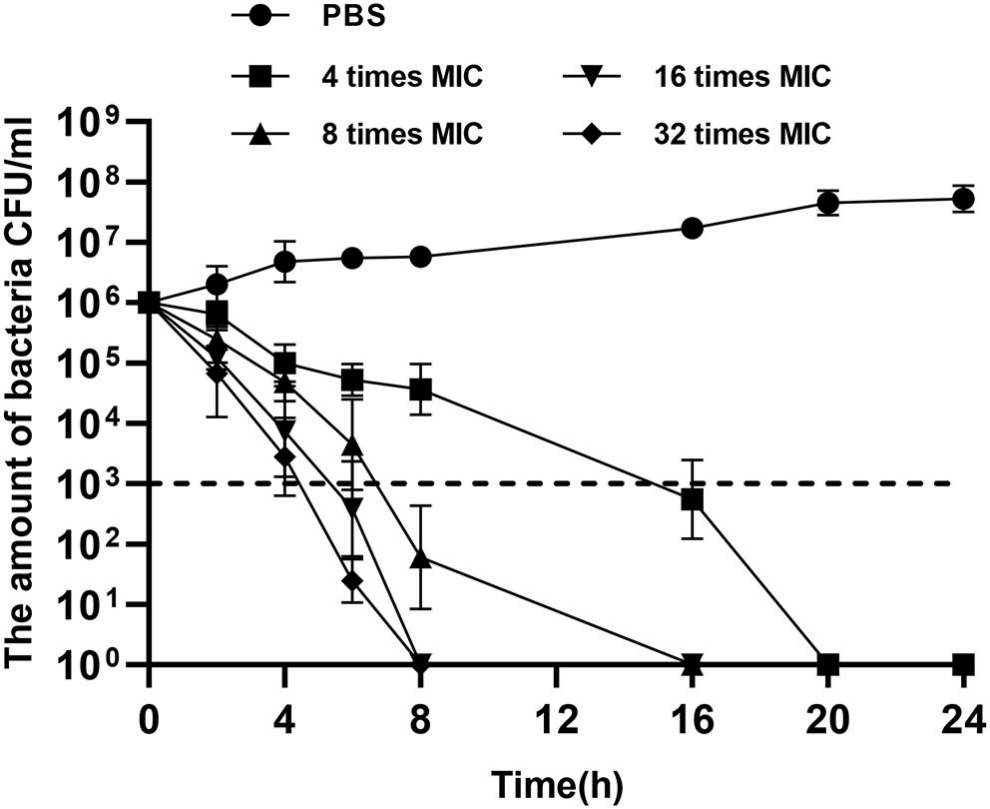
Minimum bactericidal concentrations (MBCs) of phillygenin against *H. pylori*.

A total of 12 components of *F. suspensa* were screened, among which phillygenin had the best antibacterial effect. The liquid chromatogram of phillygenin is displayed in Figure 1. The MIC of phillygenin against *H. pylori* was 16–32 μg/ml, and the MICs of other components were all >128 μg/ml, as illustrated in Table 1. The inhibition effects of phillygenin on 18 *H. pylori* strains were tested, in which it was found to have a good inhibitory effect on sensitive, drug-resistant, and multiple-resistant strains with the MIC being 16–32 μg/ml, as displayed in Table 2. The MBC of phillygenin against *H. pylori* was 16 times the MIC in the normal medium (PH 7.0), with the antibacterial rate reaching 99.9% after 6 h and 99.999% after 8 h. The antibacterial rate was 90, 99, and 99.9% with phillygenin at a concentration of 8 times the MIC for 4, 6, and 8 h in a dose-and time-dependent manner, as illustrated in Figure 2.

**Table 1:** Minimum inhibitory concentrations (MICs) of 12 monomer components of *Forsythia suspensa* against *Helicobacter pylori* (μg/ml).

**Ingredient**	**26,695**	**HPBS001**
Phillygenin	32	16
Phillyrin	>128	>128
Phillygenin A	>128	>128
Esculin	>128	>128
Arctigenin	>128	>128
Wedelolactone	>128	>128
Demethylwedelolactone	>128	>128
Mycoporphyrin	>128	>128
Phillygenin E	>128	>128
Phillygenin F	>128	>128
Isoforsythiaside	>128	>128
(+)-Pinoresin-β-	>128	>128
D-glucopyranoside

*26,695 is a sensitive strain; HPBS001 is a resistant strain (resistant to levofloxacin, clarithromycin, and metronidazole)*.

**Table 2:** The MICs of phillygenin against *H. pylori* (μg/ml).

**Strain**	**Drug resistance**	**Phillygenin**
26695	Sensitive	32
G27	Sensitive	16
MSD132	Sensitive	16
NSH57	Sensitive	32
HPBS001	Resistant to LEV, CLA, and MET	16
HPBS002	Resistant to MET	16
HPBS003	Resistant to CLA	16
HPBS004	Resistant to LEV	32
HPBS005	Resistant to LEV and LEV	32
HPBS006	Resistant to CLA and MET	16
HPBS007	Resistant to CLA	32
HPBS010	Resistant to MET, CLA, and LEV	16
HPBS011	Resistant to MET and CLA	16
HPBS012	Sensitive	32
HPBS013	Resistant to MET, CLA, and LEV	16
HPBS014	Resistant to MET, CLA, AMO, and LEV	16
HPBS015	Sensitive	16
HPBS016	Sensitive	16

*The abbreviations for antibiotics are as follows, LEV, levofloxacin; CLA, clarithromycin; MET, metronidazole; AMO, amoxicillin*.

### Antibacterial Effect of Phillygenin *in vivo*

**Figure 3:**
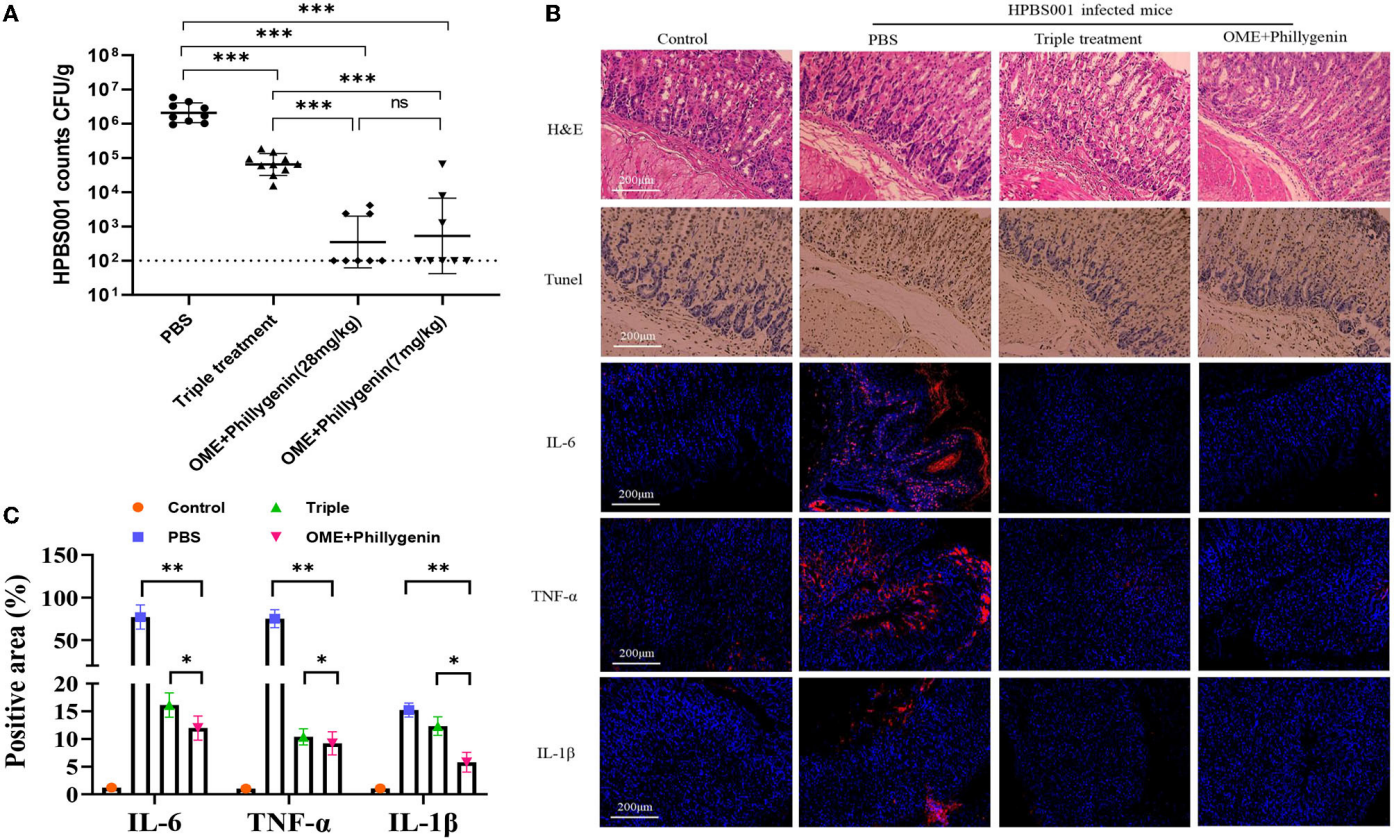
Evaluation of the antibacterial effect of phillygenin in mice. **(A)** Presents the amount of HPBS001 colonization of mice with acute gastritis; **(B)** displays the repair of gastric mucosal tissues of mice with acute gastritis; and **(C)** shows the quantitative graph of inflammatory factors. ns *P* > 0.05, *P < 0.05, **P < 0.01, ***P < 0.001.

The efficacy of phillygenin on *H. pylori in vivo* was evaluated by the acute gastritis mice models, which had been confirmed to be infected with *H. pylori* (HPBS001). According to the counted amount of colonization, the antibacterial effect of phillygenin was better than that of the triple therapy with no significant difference between high and low concentrations, as displayed in Figure 3A. The H&E staining and immunohistochemical images of the phillygenin group showed that the number of the apoptotic cells in the gastric mucosae of the mice and that of the inflammatory factors were significantly decreased (Figure 3B). In addition, the expressions of several inflammatory factors in the samples of gastric tissues were detected, with results revealing that the expression levels of interleukin (IL)-6, tumor necrosis factor (TNF)-α, and IL-1β were the lowest in the phillygenin group, as shown in Figures 3B,C, which suggested that phillygenin had a good antibacterial effect *in vivo*.

### Safety Evaluation of Phillygenin

**Figure 4:**
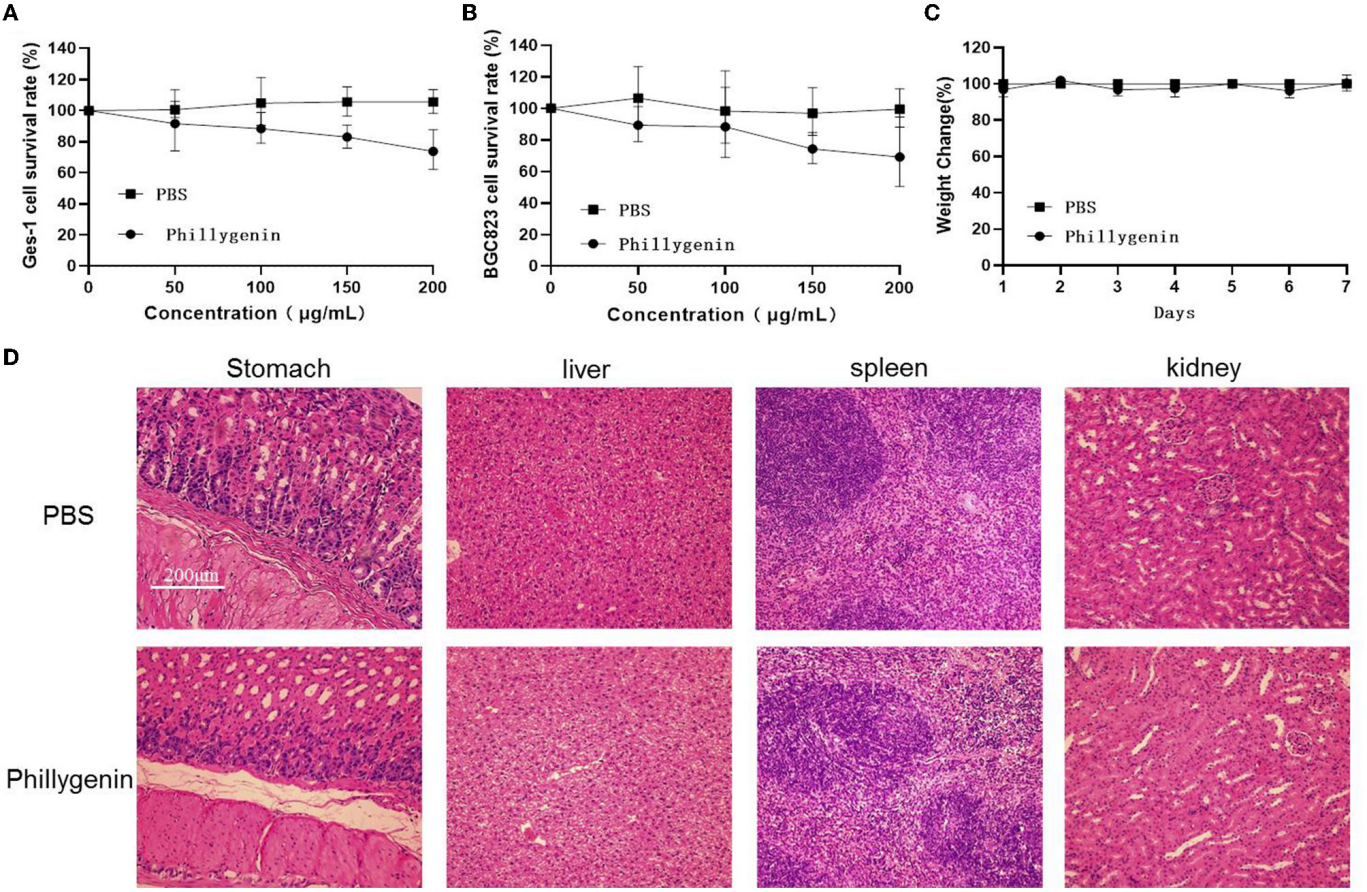
Phillygenin toxicity test. **(A)** The phillygenin toxicity test for gastric epithelial cell (GES)-1 cells; **(B)** the phillygenin cytotoxicity test for BGC823; **(C)** the effect of phillygenin on the weights of mice; and **(D)** the detection of the damage of phillygenin to stomachs, livers, spleens, and kidneys in mice.

The toxicity test of phillygenin was carried out. The results showed that phillygenin at 100 μg/ml had no obvious cytotoxic effect on GES-1 and gastric cancer cells BGC823, with a survival rate above 90% (Figures 4A,B). After the intragastric administration of 10 times the therapeutic dose of phillygenin, there was no significant change in the weights of mice within 7 days, as shown in Figure 4C. Besides, no obvious pathological damage was found in the stomachs, livers, spleens, and kidneys of mice, as shown in Figure 4D. Phillygenin that was found to have a low toxicity *in vitro* and *in vivo* and high safety could be used as the first-line therapy for *H. pylori*.

### Advantages of Phillygenin

**Figure 5:**
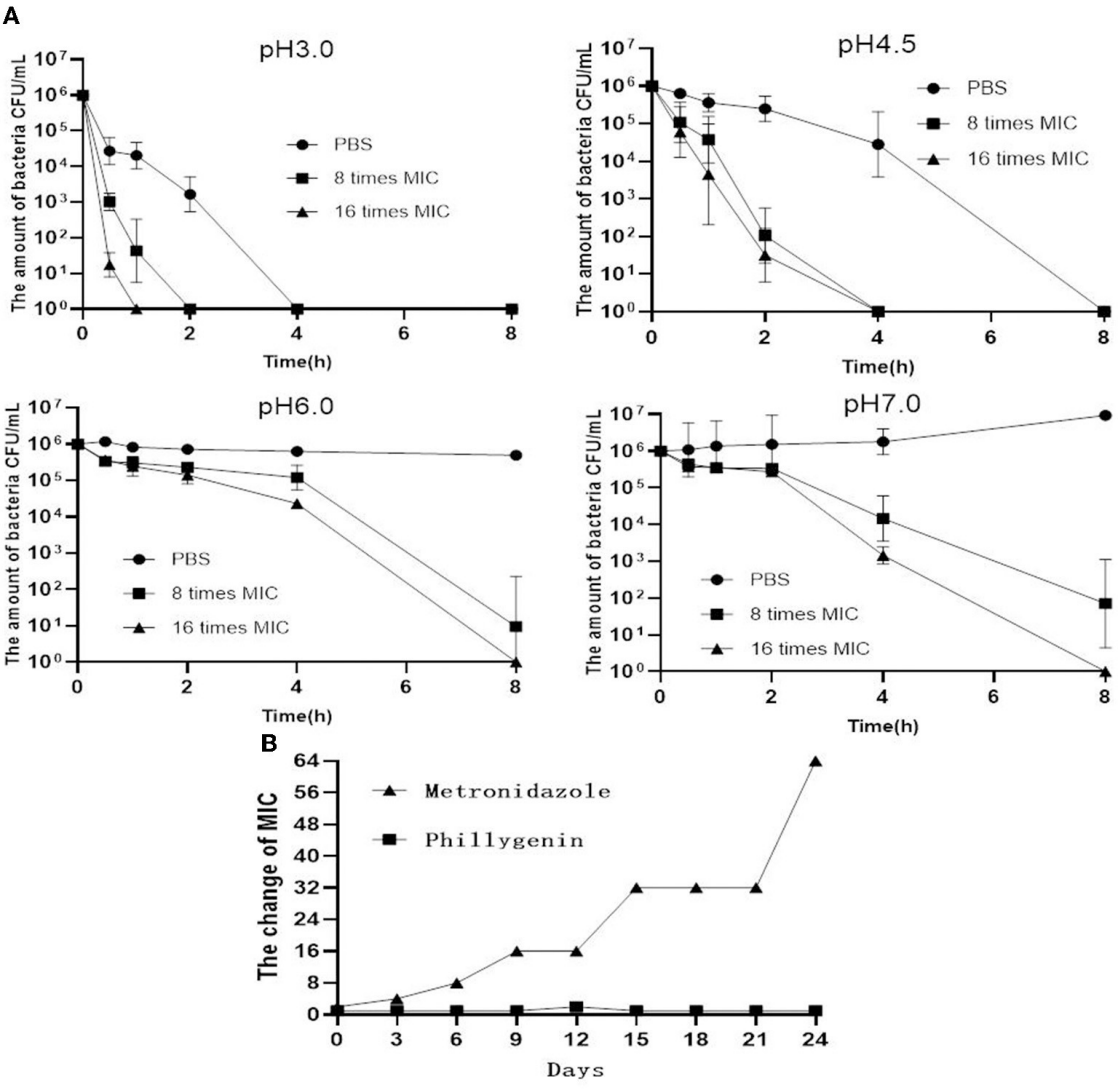
Acid response and drug resistance induction of phillygenin. **(A)** The detection of MBCs of phillygenin (μg/ml) against *H. pylori* at different pHs and **(B)** the detection of inducing drug-resistant *H. pylori* to phillygenin.

To check the selectivity of phillygenin against *H. pylori*, a total of 20 non-*H. pylori* strains were used and showed MICs all >128 μg/ml. Phillygenin that was found to have a single effect on *H. pylori* (Supplementary Table 3) was a narrow-spectrum antibiotic with a specificity and not much impact on other microflora. The inhibitory effects of phillygenin under different pH conditions (i.e., 3.0, 4.5, 6.0, and 7.0) were explored for it exerted effects after entering the stomach under low pH conditions, as shown in Figure 5A. At pH 3.0 and 4.5, the antibacterial rate reached 99.9% 2 h after the administration of 8 times and 16 times MICs of phillygenin, which could not be found at pH 6.0 and 7.0. These results indicated that phillygenin were more effective in an acidic environment with acid resistance. The drug resistance induction of phillygenin on *H. pylori* G27 strains was detected (Figure 5B). The results showed that during the 24 days drug resistance induction, the MIC of phillygenin only doubled on the 12th day with no change at other times, while the MIC of metronidazole increased by 64 times. Phillygenin that was shown to have difficulty in making *H. pylori* develop resistance could be used for clinical treatment.

### Inhibition of Phillygenin on Biofilms and ATP

**Figure 6:**
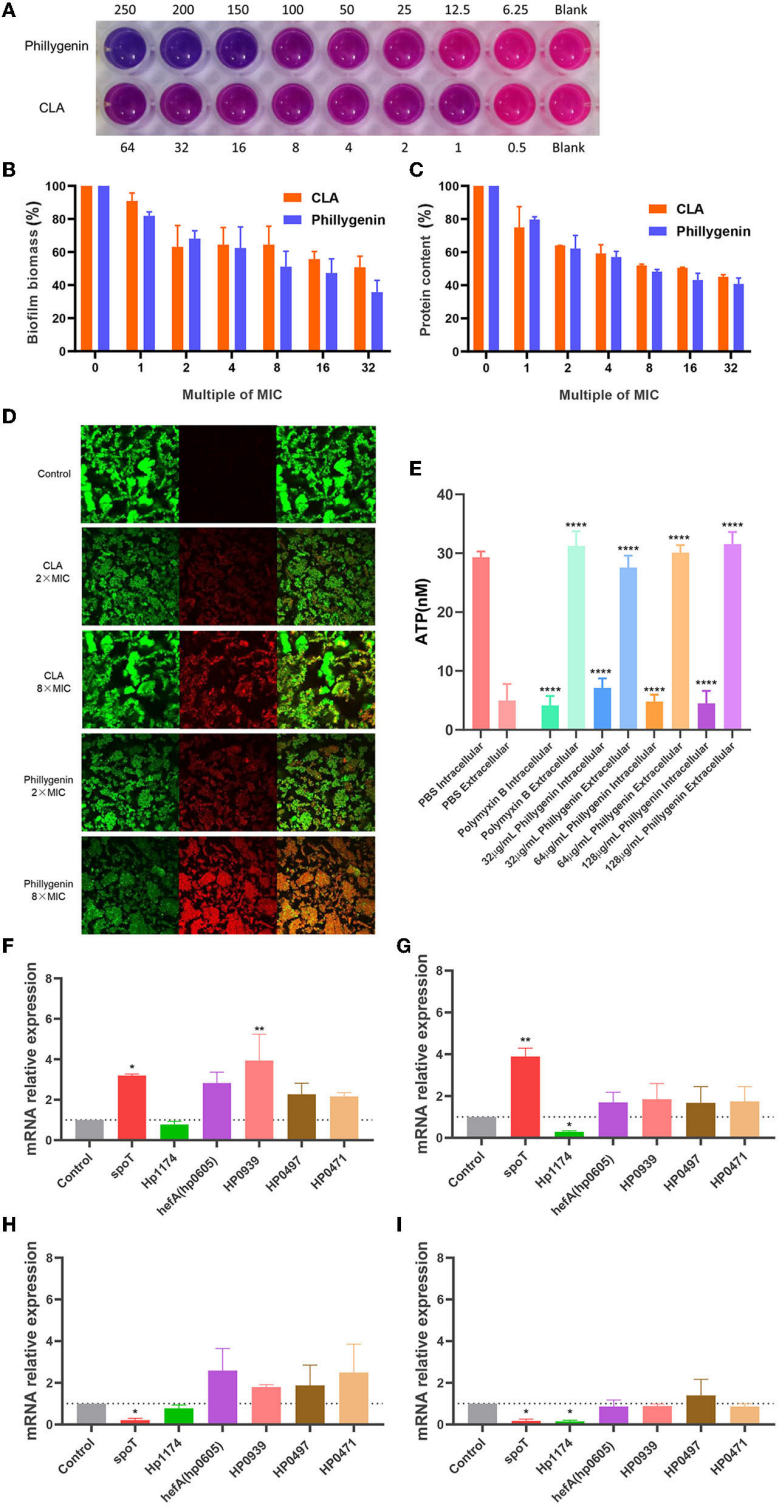
The inhibitory effect of phillygenin on biofilms and adenosine triphosphate (ATP). **(A)** The detection of the inhibition of biofilms using the Alamar blue assay; **(B)** the detection of biofilm expression levels through the crystal violet staining; **(C)** the expression levels of biofilm protein; **(D)** the detection of biofilm through the confocal laser scanning microscopy at a 400 magnification; **(E)** ATP detection; **(F)** the changes in the relative expression quantity of the mRNA of biofilm-related genes in suspended bacteria treated with phillygenin at a concentration of two times of the MIC; **(G)** the changes in the relative expression quantity of the mRNA of genes in planktonic bacteria treated with phillygenin at a concentration of four times of the MIC; **(H)** the changes in the relative expression quantity of the mRNA of biofilm-related genes in bacteria of biofilms treated with phillygenin at a concentration of two times of the MIC; **(I)** the changes in the relative expression quantity of the mRNA of biofilm-related genes in bacteria of biofilms treated with phillygenin at a concentration of four times of the MIC. *P < 0.05, **P < 0.01, ***P < 0.001.

The formation of biofilms is an important cause for the drug resistance of *H. pylori* (Krzyzek et al., 2021). Phillygenin was shown to inhibit biofilms of *H. pylori* at 100–150 μg/ml (about 6–8 times MIC) through a detection employing the Alamar blue assay (Figure 6A). Besides, it was found through the crystal violet staining assay that a drug concentration eight times of the MIC could inhibit 50% of the biofilm growth, with an effect better than that of clarithromycin (Figure 6B). Since the main component of biofilm is protein, the detection of the protein content of the biofilms of *H. pylori* was carried out. The results showed that a drug concentration 8 times of the MIC could inhibit 50% of the protein in biofilms, which was consistent with the results of the crystal violet staining and Alamar blue assay, as shown in Figure 6C. Phillygenin at a concentration 8 times of the MIC could destroy biofilms of *H. pylori* as shown in Figure 6D photographed by the confocal microscope at a 400 magnification.

ATP serves as the direct energy source for life activities and changes in levels of ATP will affect functions of cell. After the effect of phillygenin on *H. pylori* for 2 h, the intracellular ATP gradually leaked to the outside of the cells with most thereof leaking at a drug concentration of two times of the MIC. However, almost all the ATP leaked at both the drug concentrations of four and eight times of the MIC. The degree of leakage was equivalent to that of polymyxin B in a dose- and time-dependent manner (Figure 6E).

The detection of the expression of biofilm-related genes was mainly conducted by detecting the differential expressions of phillygenin on suspended bacteria and bacteria biofilms at 0 h and 4 h. The effect of a drug concentration two times of the MIC on suspended bacteria was shown in Figure 6F; the effect of a drug concentration four times of the MIC on suspended bacteria was displayed in Figure 6G. After drug action, the *Hp1174* genes that formed through biofilm regulation were significantly downregulated, with the remaining ones upregulated. The effect of a drug concentration two times of the MIC on the bacteria in biofilms was as shown in Figure 6H and that of a drug concentration four times of the MIC on the bacteria in biofilms was displayed in Figure 6I. After the drug action, the *spoT* genes and *Hp1174* genes that regulated the formation of biofilms were significantly downregulated; after the effects of phillygenin at the drug concentrations two times and four times of the MIC, there was no significant change in *hefA, HP0939, HP0497*, and *HP0471*. The *Hp1174* genes involved in the biofilm formation, whether in the suspension or bacteria in biofilms, were significantly downregulated after drug actions at different concentrations. Therefore, it could be inferred that phillygenin might inhibit biofilm formation mainly through the downregulation of *Hp1174* genes. It is noteworthy that in planktonic bacteria, *spoT* genes were upregulated, while *spoT* genes in biofilm bacteria were downregulated, which might be attributed to the characteristics of the bifunctional hydrolase in *spoT* (Bahareh et al., 2017): guanosine tetraphosphate ((p)ppGpp) that was the key to bacterial biofilm formation could be both hydrolyzed and promoted; therefore, it could be inferred that in suspended bacteria, *spoT* might hydrolyze (p)ppGpp and promote the generation thereof after biofilm formation (Cai et al., 2020).

### Transcriptome Sequencing

**Figure 7:**
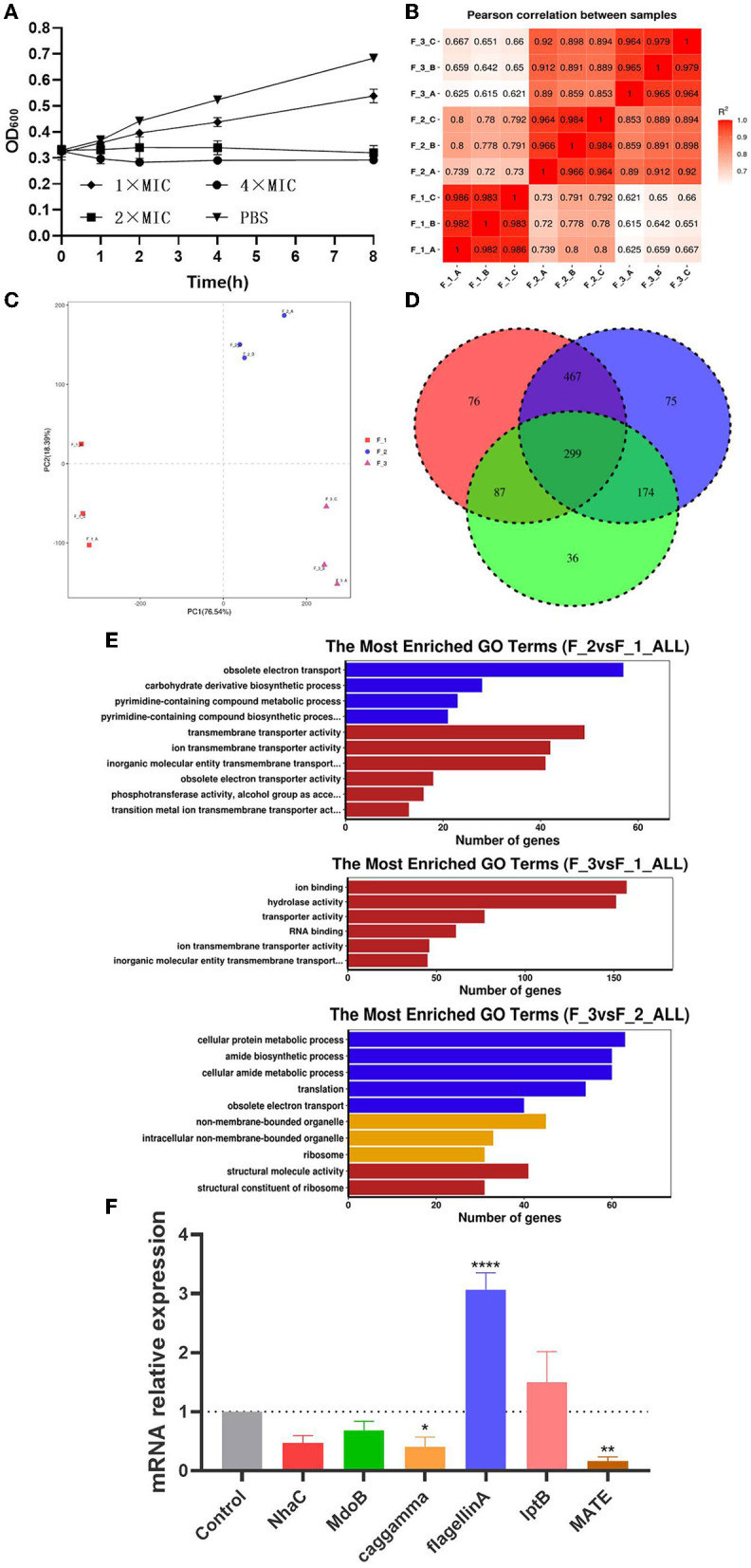
Phillygenin inhibiting *Helicobacter pylori* by Transcriptome analysis. **(A)** The half inhibitory concentration curve; **(B)** the RNA-Seq correlation analysis; **(C)** the principal component analysis (PCA); **(D)** volcano (red represents the upregulation, green represents the downregulation, and blue represents no change); **(E)** the gene ontology (GO) enrichment analysis histogram of differentially expressed genes (blue represents biological processes, yellow represents the cellular component, and red represents the molecular function); **(F)** the changes in the relative expression quantity of the mRNA of differentially expressed genes detected by the transcriptome sequencing in suspended bacteria treated with phillygenin at a concentration of two times of the MIC. *P < 0.05, **P < 0.01, ***P < 0.001.

In the proposed transcriptome sequencing, the drug action of phillygenin at different times was first detected, and the curves of half inhibitory concentrations were drawn as shown in Figure 7A, whereas, there was no change in the OD value at a drug concentration of 2 times of the MIC. The RNA-seq correlation analysis of the results showed a good biological repeatability (Figure 7B). Using the PCA, differences were shown between groups (Figure 7C). Through a pairwise comparison, 929 differential genes were detected between F_1 group and F_2 group, of which 445 were upregulated and 484 were downregulated; 1,015 differential genes were detected between F_1 group and F_3 group, of which 467 were upregulated and 548 were downregulated; 596 differential genes were detected between F_2 group and F_3 group, of which 315 were upregulated and 281 were downregulated. In the Venn diagram of differentially expressed genes, it was shown that there were 299 repetitive differentially expressed genes among three groups (Figure 7D). The differentially expressed genes were drawn into a gene ontology (GO) enrichment analysis histogram and divided into three categories, namely, biological processes, cellular components, and molecular functions. The differential genes between each group were found to mostly concentrate on pathways such as obsolete electron transport, transporter activity, ion binding, etc. (Figure 7E). According to the correlation between groups (log 2-fold change), and the comparison of repeated genes between groups to the exclusion of unknown proteins, six repeated differentially expressed genes that were most relevant were screened (including two upregulated and four downregulated ones), as shown in Table 3. According to the results of Kyoto Encyclopedia of Genes and Genomes (KEGG) enrichment and GO analysis, the differential genes mainly concentrated on the pathway for efflux transport, which was further verified by RT-qPCR to compare the differential expression levels of the effect of phillygenin on suspended bacteria with 0 h and 8 h, as shown in Figure 7F. *Nhac, caggamma, MATE*, and *MdoB* genes were downregulated, and *flagellin A* and *lptB* genes were upregulated, which were consistent with the results of the transcriptome sequencing.

**Table 3:** Differentially expressed genes.

**Gene**	**Name**	**Log2 fold change**	**Description**	**Enrichment pathway**
*Nhac*	HPG27_RS04635	−7.1404	Sodium:protonantiporter	The inorganic molecular entity transmembrane transporter
				activity
*Caggamma*	HPG27_RS02515	−5.0855	Sodium:calciumantiporter	The epithelial cell signaling in Helicobacter pylori infection
*MATE*	HPG27_RS03695	−5.9103	MATEfamilyeffluxtransporter	The transmembrane transporter activity
*MdoB*	HPG27_RS02805	−7.4155	LTAsynthasefamilyprotein	The sulfuric ester hydrolase activity
*flagellinA*	HPG27_RS02925	4.0196	FlagellinA	The two-component system/Flagellar assembly
*lptB*	HPG27_RS03465	5.3738	LPSexportABCtransporterATP-bindingprotein	The ABC transporters

## Discussion

*Helicobacter pylori* is a cause for the occurrence of various intestinal and extra-intestinal diseases and severe drug resistance (Sugano et al., 2015; Okuda et al., 2016). The development of new antibiotics is an important way to solve *H. pylori*'s drug resistance, and screening of effective ingredients from natural products is a convenient method for new drug development. In this study, phillygenin, which is an effective antibacterial component of *F. suspensa* belonging to the lignans, was screened out (Liu et al., 2018). Studies have reported that phillygenin had the effects of regulating the intestinal microbiota, reducing fibrosis herein (Sun et al., 2021), inhibiting the release of inflammatory cytokines (Wang et al., 2021), inhibiting adhesion and migration, etc. (Quan et al., 2021), while, less has been studied about its antibacterial effects.

It was observed that phillygenin has the same effect on both sensitive and drug-resistant *H. pylori* strains. The effect was a concentration- and time-dependent. In the efficacy evaluation *in vitro*, the MICs of phillygenin against 20 non-*H. pylori* strains were detected, and phillygenin was found to have a specific inhibitory effect on *H. pylori*. In the CCK-8 cytotoxicity test, the survival rates of GES-1 and BGC823 cells were above 90% with phillygenin at a concentration of 100 μg/ml (6.25 times MIC); in addition, after the intragastric administration of 10 times of the therapeutic dose to mice, no damage was found in the organs; therefore, phillygenin was found to have a high safety through the *in vivo* and *in vitro*. In the efficacy evaluation *in vivo*, the treatment of mice infected with HPBS001 (resistant to levofloxacin, clarithromycin, and metronidazole) using phillygenin, its antibacterial effect was found to be better than that of the triple therapy; therefore, it could be suggested that phillygenin which exerted a good therapeutic effect on drug-resistant strains *in vivo* and on refractory gastritis caused by clinically drug-resistant *H. pylori* infection, can serve as a lead drug or candidate drug for treating *H. pylori*.

The long-term use of antibiotics in general is more prone to rendering *H. pylori* drug-tolerant, but the degrees of drug resistance vary among different antibiotics (Lu et al., 2020). In the comparison of phillygenin and MET, no obvious drug resistance to phillygenin was found after 24 days of drug resistance induction with the MIC of phillygenin increasing by two times and that of metronidazole by 64 times. It could be suggested that phillygenin had difficulty in rendering *H. pylori* drug resistant, which might be attributed to its plant origin and multiple targets. The discovery of this strength, whereas, may lay the foundation for increasing the dosage or usage of phillygenin in the treatment of *H. pylori* in the future and help this compound become a lead drug. On the contrary, oral drugs were found to fail to achieve the desired effect due to the low pH environment in gastric juice (Li et al., 2021), while the antibacterial rates of phillygenin at the concentrations of eight times and 16 times of the MIC at pH 3.0 and 4.5 reached 99.9% after 2 h of administration, an antibacterial rate that was not observed at pH 6.0 and 7.0. It could be suggested that phillygenin exerted a better antibacterial effect under low pH conditions and resisted the acidic environment in the stomach, which was its another advantage. The *Nhac* genes mainly regulated the transport of Na^+^/H^+^, which helped *H. pylori* better colonize in the acidic environment (Ge et al., 2018). However, phillygenin could downregulate the *Nhac* genes and exert better effect in the acidic environment.

At present, the international competition of drug research is mainly on the research of drug targets. Generally speaking, a new target of drug action once discovered will tend to become a breakthrough in the discovery of a series of new drugs (Wu et al., 2021). The bacterial biofilm is a bacterial community called extracellular polymer (EPS) in a self-assembled matrix, and this produced by *H. pylori* is mainly composed of proteins (Hathroubi et al., 2018). The bacterial biofilm once formed becomes a refuge for bacteria to resist the antibiotic treatment and immune defense, which is also referred to as the development of drug resistance (Rizzato et al., 2019). Guanosine tetraphosphate (p-ppGpp) can affect the formation of bacterial biofilms. *SpoT* is a bifunctional enzyme with the properties of p-ppGpp synthase and hydrolase (Hathroubi et al., 2017). It is noteworthy that in planktonic bacteria, spoT genes were upregulated, while *spoT* genes in biofilm bacteria were downregulated, which might be attributed to the characteristics of the bifunctional hydrolase in *spoT* (Bury-Moné et al., 2004): guanosine tetraphosphate ((p)ppGpp) that was the key to bacterial biofilm formation could be both hydrolyzed and promoted; therefore, it could be inferred that in suspended bacteria, *SpoT* might hydrolyze (p)ppGpp and promote the generation thereof after biofilm formation (Wang et al., 2011). The efflux pump is also one of the mechanisms leading to the antimicrobial properties of biofilms (Atkinson et al., 2011). *Hp1174* is a gene of the major facilitator superfamily (MFS) efflux pump family. Studies have found that *spoT* and *Hp1174* genes were involved in the formation of biofilms (De-Kievit et al., 2001). The phillygenin alluded to in this research was found to inhibit the formation of biofilms and downregulate the *spoT* and *Hp1174* genes that regulated biofilm formation. *SpoT, Hp1174, lptB, Nhac*, and *MATE* genes are all transmembrane proteins, indicating that the mechanism of phillygenin might also inhibit the formation of biofilms and change the permeability of the membranes. The *Caggamma (Cag4)* gene, which is a lytic transglycosylase encoded by the Cag pathogenicity island, can hydrolyze peptidoglycan layer of bacteria, release intracellular proteins into the periplasmic space, promote the assembly and formation of the IV secretion system, help the host evade immune detection, and contribute to the long-term colonization of bacteria (Lai et al., 2017). Phillygenin was shown to downregulate the Caggamma genes and prevent bacterial colonization. The spatial organization of the population, such as biofilm, was found to increase the production of some virulence factors (Ge et al., 2018); therefore, phillygenin that downregulated the *Caggamma* genes of the virulence factors could partly confirm the inhibitory effect of phillygenin on biofilms.

ATP was found to provide energy in many key cellular processes and reactions (Quinn et al., 2021). The decrease of ATP level suggested that the functions of mitochondria were impaired, and the cells would therefore undergo apoptosis and necrosis (Arya et al., 2019). The Lpt protein family was found to be required in the export of lipopolysaccharide (LPS) to the cell surface (Turkina et al., 2015). The results of this study suggested that the drug action of phillygenin on *H. pylori* would cause the leakage of intracellular ATP, the degree of which was positively correlated with the concentration of phillygenin. This might be associated with the upregulation of the lptB genes (Sperandeo et al., 2007). The *LptB* gene was found to bind to the transmembrane *LptFG* complex on the cytoplasmic side to hydrolyze ATP, thereby providing energy to accelerate the transport of ATP (Martorana et al., 2016). The leakage of ATP alluded to in this research, which might be associated with the upregulation of the *LptB* genes by phillygenin, was consistent in the phenotype with those of the ATP metabolism, acid resistance, and inhibition of biofilm formation in the above.

In the transcriptome sequencing, it was found that the drug action of phillygenin on *H. pylori, Nhac, MATE*, and *MdoB* genes was downregulated and *flagellin A* genes upregulated. The *MATE* genes, which were found to mainly regulate the efflux of drugs, could be significantly downregulated by phillygenin to decrease the efflux of drugs. The *MdoB* gene, which is a kind of DNA methyltransferase, is involved in DNA methylation (Tan et al., 2016). The downregulation of this gene, however, indicates the damage of DNA and cell death. The *Flagellin A (FlaA)* gene not only regulates the composition of mastigoneme but is also one of the main antigens that produce the serum IgG and gastrointestinal IgA (Zarei et al., 2017). The upregulation of *FlaA* genes, however, may be associated with the immune response produced by the body. In addition, the antiadhesion effects and *in vivo* oxidation (ROS) were studied, both of which did not work effectively at low concentrations but worked effectively under high concentrations, as show in Supplementary Figures 1, 2. Although biofilm-related genes were not preferentially revealed in transcriptome sequencing, this may be related to the drug action time because the strains extracted by transcriptome sequencing were treated with drugs for 8 h, while the strains extracted during biofilm-related gene detection were treated with drugs for 4 h.

## Conclusion and Outlook

**Figure 8:**
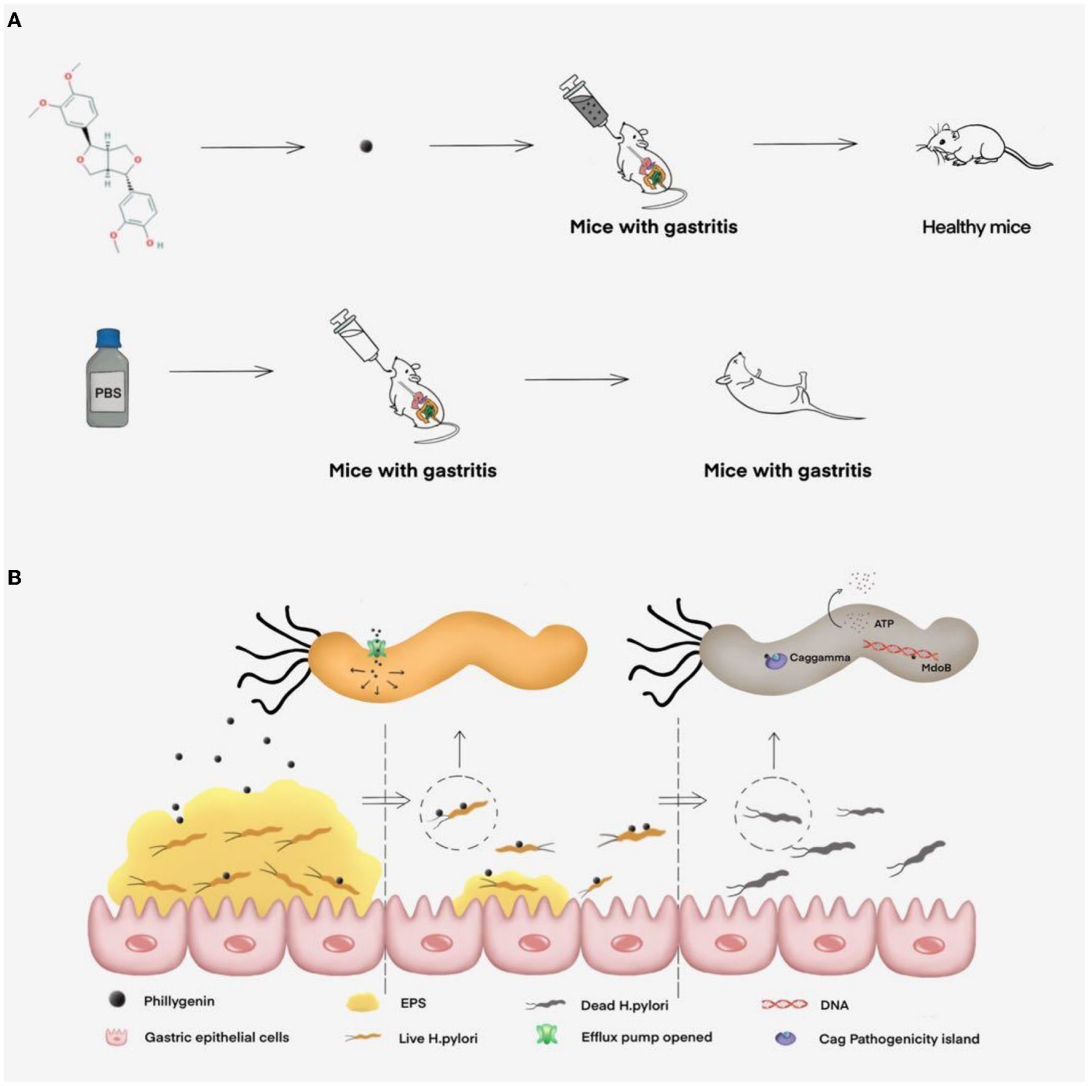
The inhibitory effect of phillygenin on *H. pylori* and its mechanism map. **(A)** The therapeutic effect of phillygenin on *H. pylori*-infected acute gastritis in mice and **(B)** the mechanism of phillygenin, mainly including inhibiting biofilm formation, causing ATP leakage, weakening *H. pylori*'s virulence, etc.

Phillygenin has good antibacterial effects *in vivo* and *in vitro* by causing ATP leakage and inhibit the biofilm formation (Figure 8), but its mechanism of action is multiple targets and pathways, which require further experimental exploration. In addition, phillygenin has the advantage of low toxicity, a difficulty in making *H. pylori* form a drug resistance, and a specific drug action on *H. pylori* and is a drug with great application potential. This study can provide experimental basis for phillygenin-inhibiting *H. pylori* and ideas for the clinical treatment of *H. pylori* and development of new drugs.

## Data Availability Statement

The datasets presented in this study can be found in online repositories. The names of the repository/repositories and accession number(s) can be found below: NCBI; PRJNA802695.

## Ethics Statement

The animal study was reviewed and approved by SYXK Gui 2017-0004.

## Author Contributions

R-JL was responsible for the experimental research. CQ, G-RH, and L-JL performed to consult literature and write the first draft. X-QM and Y-QH designed, checked, modified, and finalized the manuscript. All authors proofed the revised manuscript. All authors contributed to the article and approved the submitted version.

## Funding

This study was supported by National Natural Science Foundation of China, Nos. 81760739 and 32060018 and through special fund projects for Guide Local Science and Technology Development by the China Government (GUIKEZY20198004).

## Conflict of Interest

The authors declare that the research was conducted in the absence of any commercial or financial relationships that could be construed as a potential conflict of interest.

## Publisher's Note

All claims expressed in this article are solely those of the authors and do not necessarily represent those of their affiliated organizations, or those of the publisher, the editors and the reviewers. Any product that may be evaluated in this article, or claim that may be made by its manufacturer, is not guaranteed or endorsed by the publisher.
