# Supplementary material for: Phillygenin Inhibits Helicobacter pylori by Preventing Biofilm Formation and Inducing ATP Leakage
Source: Front Microbiol. 2022 Apr 28;13:863624. doi: 10.3389/fmicb.2022.863624 (PMC9097866; doi:10.3389/fmicb.2022.863624)
Supplement: Supplementary file 1 [file Data_Sheet_1.docx]

Table 1 Strain information

| Strains | Origin | Medium composition | Culture temperature |
| --- | --- | --- | --- |
| Proteus mirabilis | Clinical separation | Nutrient Agar/ Broth | 37° |
| Cryptoccus neo formans | Clinical separation | Sabourauds | 30° |
| Candida tropicalis | Clinical separation | Sabourauds | 37° |
| Campylobacter | Clinical separation | Columbia Agar Base / Brain-Heart Infusion Broth（With 10% serum） | 3 7° |
| Bacillus subtilis | Clinical separation | Sabourauds | 37° |
| Morganella morganii | Guangdong Microbial Culture Collection Center | Nutrient Agar/ Broth | 37° |
| Staphylococcus haemolyticus | Clinical separation | Nutrient Agar/ Broth | 37° |
| Stenotrophomonas maltophilia | Clinical separation | Nutrient Agar/ Broth | 30° |
| Acetobacter pasteurianus | Guangdong Microbial Culture Collection Center | Glucose、Radix scutellariate、Calcium carbonate、Ethanol absolute、Distilled water、Agar（No need to liquid medium） | 30° |
| Escherichia coli | Clinical separation | Nutrient Agar/ Broth | 37° |
| Lactobacillus curvatus | Guangdong Microbial Culture Collection Center | Peptones、Beef Extract、Yeast extract、Glucose、Sodium acetate、Ammonium citrate dibasic、tween-80、Dipotassium hydrogenphosphate、magnesium sulfate heptahydrate、Manganese sulfate heptahydrate、Calcium carbonate、Distilled water、Agar（No need to liquid medium） | 37° |
| Saccharomyces cerevisiae Hansen | Guangdong Microbial Culture Collection Center | Peptone、Glucose、Yeast extract、Malt extract、Distilled water、Agar | 30° |
| b.fragilis | Guangdong Microbial Culture Collection Center | TSA+5% Defibrinated Sheep Blood | 37° |
| Bifidobacterium longum | Guangdong Microbial Culture Collection Center | BactoTM Soytone、Typtone、Yeast extract、Glucose、saline solution、L-Cysteine、0.1%resazurin、Distilled water、Agar（No need to liquid medium） | 37° |
| Enterobacterhormaechei | Clinical separation | Nutrient Agar/ Broth | 37° |
| Staphalococcus aureus | Clinical separation | Nutrient Agar/ Broth | 37° |
| candida Albicans | Clinical separation | Sabourauds | 37° |
| Klebsiella pneumoniae | Clinical separation | Nutrient Agar/ Broth | 37° |
| Pseudomonas aeruginosa | Clinical separation | Nutrient Agar/ Broth | 37° |
| Acinetobacter baumannii | Clinical separation | Nutrient Agar/ Broth | 37° |

Table 2 Primers list

| Gene | Forward primer | Reverse primer |
| --- | --- | --- |
| 16s | AGGATCAAGGTTTAAGGATT | CTGGAGAGACTAAGCCCTCC |
| spoT | TGTGCAACCTGTCGCTAATC | TTGGGCAAGATGTGGCTAAT |
| Hp1174 | AAAATGGGCGATAATGCAAG | TCCACATCAGGCAATTTCAA |
| hefA(hp0605) | CTCGCTCGCATGATCGC | CGTATTCGCTCAAATTCCCT |
| Hp0939 | CACGCCAAGCTTGAGTAACA | CAAAGCGGCTTCCAAATAAA |
| Hp0497 | TTGCTGGCATCACTTCTACG | AAATCAACACCACGCCTACC |
| Hp0471  NhaC  MdoB | TTCCCTTGTTTTAGGCATGG  AACGACGCTCATAACTCCACACG  CGAACCGTTAAGAGCCTGGATGTC | TGGGAATGGCTGCAATATCT  AGAAATAGGAACCAGCAAGCACACC  TTGCCAATCGCCATAGGAAAGCTAG |
| Caggamma | AGGCTGCGACAATGAAGTGGTG | CGCGCTTGTTGTTCAACCCTAAAG |
| FlagellinA | CGCAGTATAGATGGTCGTGGGATTG | CGTGTGAGAGAAAGCCTTCCGTAG |
| LptB | GCGAAGTGGTGGGGCTTTTAGG | ACGCTTGTGTAAGGGGTATTTGGC |
| MATE | TTATACACCGGCACTAACGCCATTC | AAGAAACAAACAGCACGCCCAAAC |

Table 3 MICs of *Phillygenin* against non- *H. pylori* strains (μg/mL)

| Strain | Drug resistance | Phillygenin |
| --- | --- | --- |
| Proteus mirabilis | Sensitive | >128 |
| Cryptoccus neo formans | Resistance | >128 |
| Candida tropicalis | Sensitive | >128 |
| Campylobacter | Sensitive | >128 |
| Bacillus subtilis | Sensitive | >128 |
| Morganella morganii | Sensitive | >128 |
| Staphylococcus haemolyticus | Sensitive | >128 |
| Stenotrophomonas maltophilia | Sensitive | >128 |
| Acetobacter pasteurianus | Sensitive | >128 |
| Escherichia coli | Sensitive | >128 |
| Lactobacillus curvatus | Sensitive | >128 |
| Saccharomyces cerevisiae Hansen | Sensitive | >128 |
| b.fragilis | Sensitive | >128 |
| Bifidobacterium longum | Sensitive | >128 |
| Enterobacterhormaechei | Sensitive | >128 |
| Staphalococcus aureus | Methicillin-resistant | >128 |
| candida Albicans | Sensitive | >128 |
| Klebsiella pneumoniae | Sensitive | >128 |
| Pseudomonas aeruginosa | Sensitive | >128 |
| Acinetobacter baumannii | Sensitive | >128 |

Table 4 Differential gene expression analysis

| GeneName | Readcount Group | readcount Group | log2FoldChange | Descrition |
| --- | --- | --- | --- | --- |
|  | F_2 | F_1 |  |  |
| HPG27_RS04420 | 181.1615717 | 7.482421219 | 4.5976 | cupindomain-containingprotein |
| HPG27_RS01105 | 7973.17251 | 330.1149895 | 4.5941 | cysteinedesulfurase%2CNifSfamily |
| HPG27_RS01220 | 82046.13986 | 3718.032398 | 4.4638 | DNAstarvation/stationaryphaseprotectionprotein |
| HPG27_RS01110 | 11042.40676 | 529.4778366 | 4.3823 | iron-sulfurclusterassemblyscaffoldproteinNifU |
| HPG27_RS03465 | 6213.14214 | 335.0706171 | 4.2128 | LPSexportABCtransporterATP-bindingprotein |
| HPG27_RS02925 | 335062.2167 | 20658.6961 | 4.0196 | S03465 |
| ansB | 4635.467342 | 322.9727438 | 3.8432 | typeIIasparaginase |
| HPG27_RS01390 | 436.7535005 | 30.65750148 | 3.8325 | ferredoxin |
| HPG27_RS01040 | 8150.244362 | 581.4286761 | 3.8092 | MRPfamilyATP-bindingprotein |
| HPG27_RS03125 | 11642.18888 | 834.1683071 | 3.8029 | NAD(P)-dependentoxidoreductase |
| HPG27_RS03695 | 16.85430521 | 375.8174357 | -4.4788 | MATEfamilyeffluxtransporter |
| HPG27_RS05095 | 13.25140619 | 300.0990922 | -4.5012 | ParAfamilyprotein |
| HPG27_RS07190 | 25.51538886 | 626.0850949 | -4.6169 | SpoIIIJ-associatedprotein |
| HPG27_RS07950 | 41.81540485 | 1037.105498 | -4.6324 | ubiquinol-cytochromeCchaperonefamilyprotein |
| HPG27_RS01250 | 9.952233968 | 289.8031601 | -4.8639 | DUF2393domain-containingprotein |
| HPG27_RS02515 | 1.039122844 | 35.28325805 | -5.0855 | sodium:calciumantiporter |
| HPG27_RS05770 | 11.07855211 | 420.3384534 | -5.2457 | endoribonucleaseYbeY |
| HPG27_RS02805 | 5.447641688 | 217.8013591 | -5.3212 | LTAsynthasefamilyprotein |
| HPG27_RS04890 | 5.789794539 | 283.6470083 | -5.6144 | proteinphosphatase |
| HPG27_RS02665 | 7.165210069 | 407.1329197 | -5.8283 | glutamateracemase |
|  | F_3 | F_1 |  |  |
| HPG27_RS01220 | 161646.5066 | 2636.201424 | 5.9382 | DNAstarvation/stationaryphaseprotectionprotein |
| HPG27_RS01555 | 38685.20404 | 823.514992 | 5.5538 | peptidoglycandeacetylase |
| HPG27_RS02925 | 622837.8723 | 14643.63654 | 5.4105 | flagellinA |
| HPG27_RS03465 | 9853.931105 | 237.6458196 | 5.3738 | LPSexportABCtransporterATP-bindingprotein |
| HPG27_RS00865 | 2948.366432 | 73.29954404 | 5.33 | molybdopterinmolybdenumtransferaseMoeA |
| HPG27_RS01105 | 9117.743603 | 233.9553579 | 5.2844 | cysteinedesulfurase%2CNifSfamily |
| HPG27_RS07225 | 34719.3099 | 1018.93961 | 5.0906 | penicillin-bindingproteinactivatorLpoB |
| HPG27_RS02305 | 689.1658675 | 21.09714138 | 5.0297 | typeIIrestrictionendonuclease |
| ansB | 7016.792739 | 229.099742 | 4.9368 | typeIIasparaginase |
| HPG27_RS00570 | 71449.74185 | 2505.12494 | 4.834 | molecularchaperoneDnaK |
| HPG27_RS02665 | 6.014791113 | 288.5717931 | -5.5843 | glutamateracemase |
| HPG27_RS03525 | 1.056869686 | 51.50340476 | -5.6068 | tRNAlysidine(34)synthetaseTilS |
| HPG27_RS04560 | 1.30035058 | 65.64953649 | -5.6578 | 7-carboxy-7-deazaguaninesynthaseQueE |
| HPG27_RS07195 | 6.634648197 | 386.3625804 | -5.8638 | tRNAuridine-5-carboxymethylaminomethyl(34)synthesisGTPaseMnmE |
| HPG27_RS03695 | 4.429486583 | 266.3913336 | -5.9103 | S02805 |
| HPG27_RS06165 | 1.828785423 | 121.8299657 | -6.0578 | DNApolymeraseIIIsubunitdelta' |
| HPG27_RS07190 | 6.413477001 | 443.6948635 | -6.1123 | SpoIIIJ-associatedprotein |
| HPG27_RS00960 | 2.733596456 | 195.9558137 | -6.1636 | phospholipaseDfamilyprotein |
| HPG27_RS04635 | 21.08302235 | 2974.521749 | -7.1404 | 02515 |
| HPG27_RS02805 | 0.904811033 | 154.470716 | -7.4155 | 02925 |
|  | F_3 | F_2 |  |  |
| HPG27_RS02980 | 114.8673088 | 13.2587847 | 3.1149 | ABCtransporterATP-bindingprotein |
| HPG27_RS02305 | 453.4100661 | 60.7982107 | 2.8987 | typeIIrestrictionendonuclease |
| HPG27_RS03845 | 17224.20718 | 2385.372382 | 2.8522 | outermembranebeta-barrelproteinHofF |
| HPG27_RS07225 | 22664.8545 | 3146.84873 | 2.8485 | penicillin-bindingproteinactivatorLpoB |
| HPG27_RS00865 | 1930.351114 | 316.1188801 | 2.6103 | molybdopterinmolybdenumtransferaseMoeA |
| HPG27_RS00140 | 1466.259481 | 253.1174175 | 2.5343 | outermembraneprotein |
| HPG27_RS02260 | 137.2051708 | 26.64118932 | 2.3646 | molybdateABCtransportersubstrate-bindingprotein |
| HPG27_RS03900 | 181.0285912 | 36.31936392 | 2.3174 | molybdopterinadenylyltransferase |
| HPG27_RS07995 | 12.27823561 | 2.464819496 | 2.3165 | MFStransporter |
| HPG27_RS01455 | 3560.824881 | 755.812295 | 2.2361 | diaminopimelatedecarboxylase |
| HPG27_RS07140 | 43.55516998 | 383.3611091 | -3.1378 | peptidylprolylisomerase |
| HPG27_RS01490 | 8.820473855 | 79.94446483 | -3.1801 | 50SribosomalproteinL27 |
| HPG27_RS06995 | 4.218889885 | 39.62250709 | -3.2314 | ribosomesilencingfactor |
| HPG27_RS07400 | 1.500641252 | 19.24037878 | -3.6805 | NifUfamilyprotein |
| HPG27_RS04060 | 4.569865564 | 77.23547672 | -4.079 | RNA-bindingprotein |
| HPG27_RS02365 | 2.078395719 | 37.33108691 | -4.1668 | 50SribosomalproteinL28 |
| HPG27_RS05725 | 0.70195136 | 13.7142528 | -4.2882 | 30SribosomalproteinS16 |
| HPG27_RS02675 | 1.8891787 | 38.47081459 | -4.3479 | 50SribosomalproteinL31 |
| HPG27_RS06730 | 3.031662545 | 68.87189773 | -4.5057 | nickel-responsivetranscriptionalregulatorNikR |
| HPG27_RS00405 | 2.618588419 | 64.05584265 | -4.6125 | 30SribosomalproteinS20 |


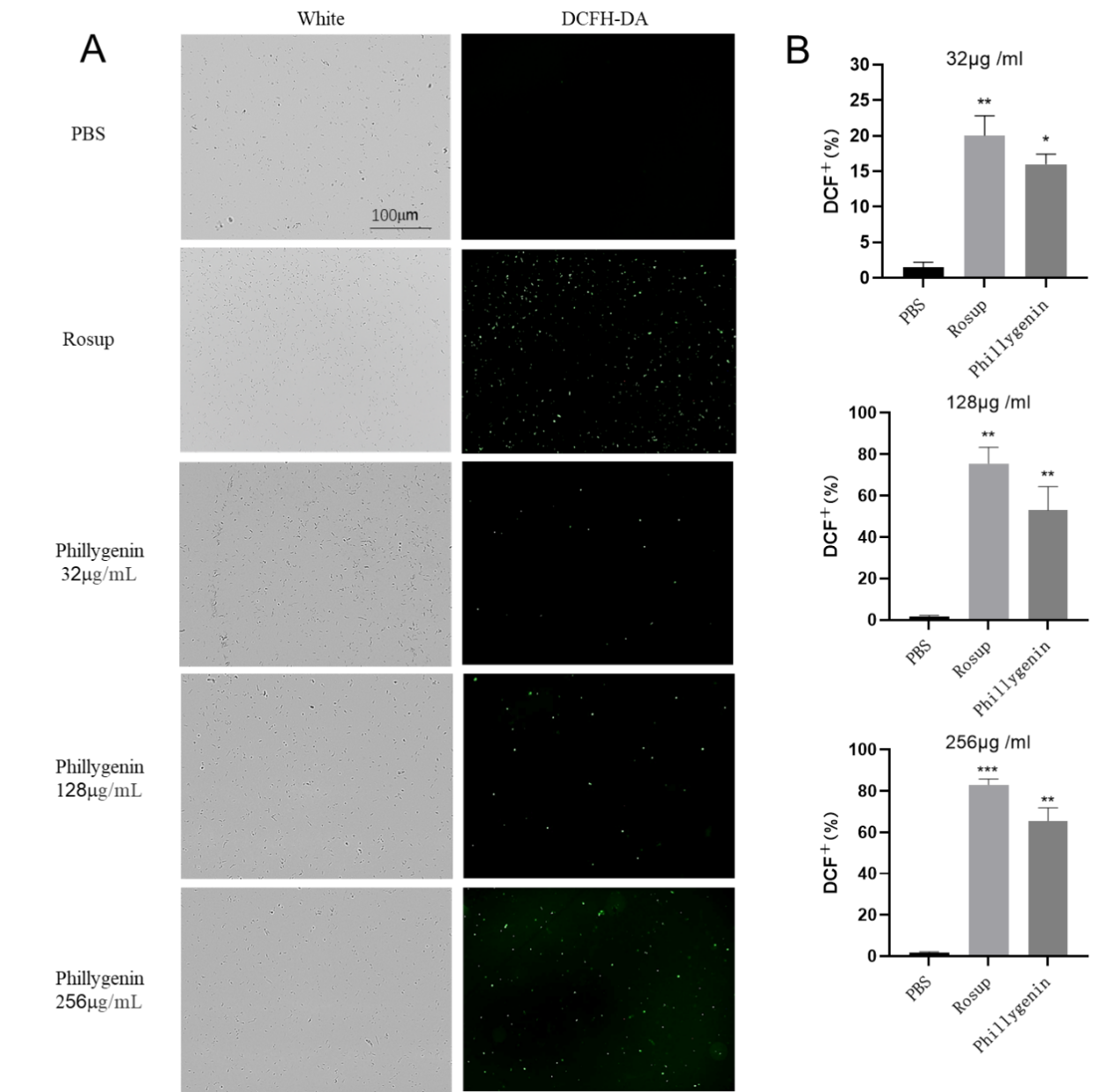


Figure 1 ROS detection.A. is the bright field and fluorescence field after drug action, taken at 400 times; the experiment uses ROS detection kit (Beyotime, China), Rosup is the positive drug control, PBS is the negative control; B. is the quantitative diagram . The results show that *Forsythiaside* penetrates into *H. pylori* cells and may undergo redox reactions with proteins, nucleic acids, lipids, etc., producing some peroxides, hydrogen peroxide, etc. These oxides can decompose DCFDA into dichlorofluorescein yellow And it produces fluorescence. Experiments have found that the oxidation reaction of forsythiaside is not strong at a low concentration of 32μg/mL, and the fluorescence produced is very weak, but the fluorescence gradually increases at 128μg/mL and 256μg/mL, so the occurrence of redox reaction is positively related to the dose of *Forsythiaside*.


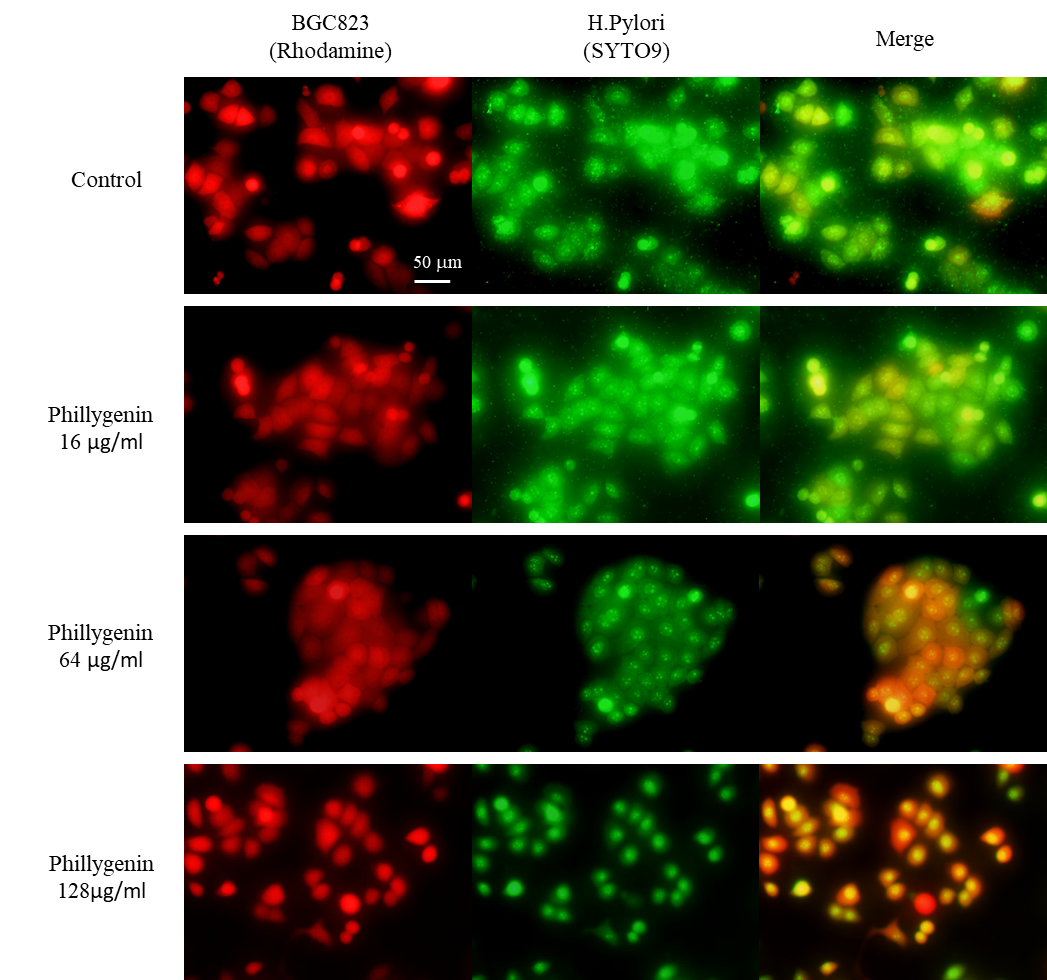


Figure 2 Bacterial adhesion cell experiment BGC823 cells were cultured in RPMI1640 medium containing 10% FBS and 1% antibiotics, the *H. pylori* suspension was collected and labeled with SYTO9 for 15 minutes, and then treated with different drug concentrations and co-cultured with the cells for 2 hours. The plate is placed under a fluorescence microscope for observation. The results showed that *Forsythiaside* was not strong in preventing cell adhesion at a low concentration of 16μg/mL, but at 64μg/mL, 128μg/mL, as the concentration increased, the bacteria gradually reduced cell adhesion. Therefore, *Forsythiaside* prevents bacteria from adhering to cells is positively correlated with the dose.


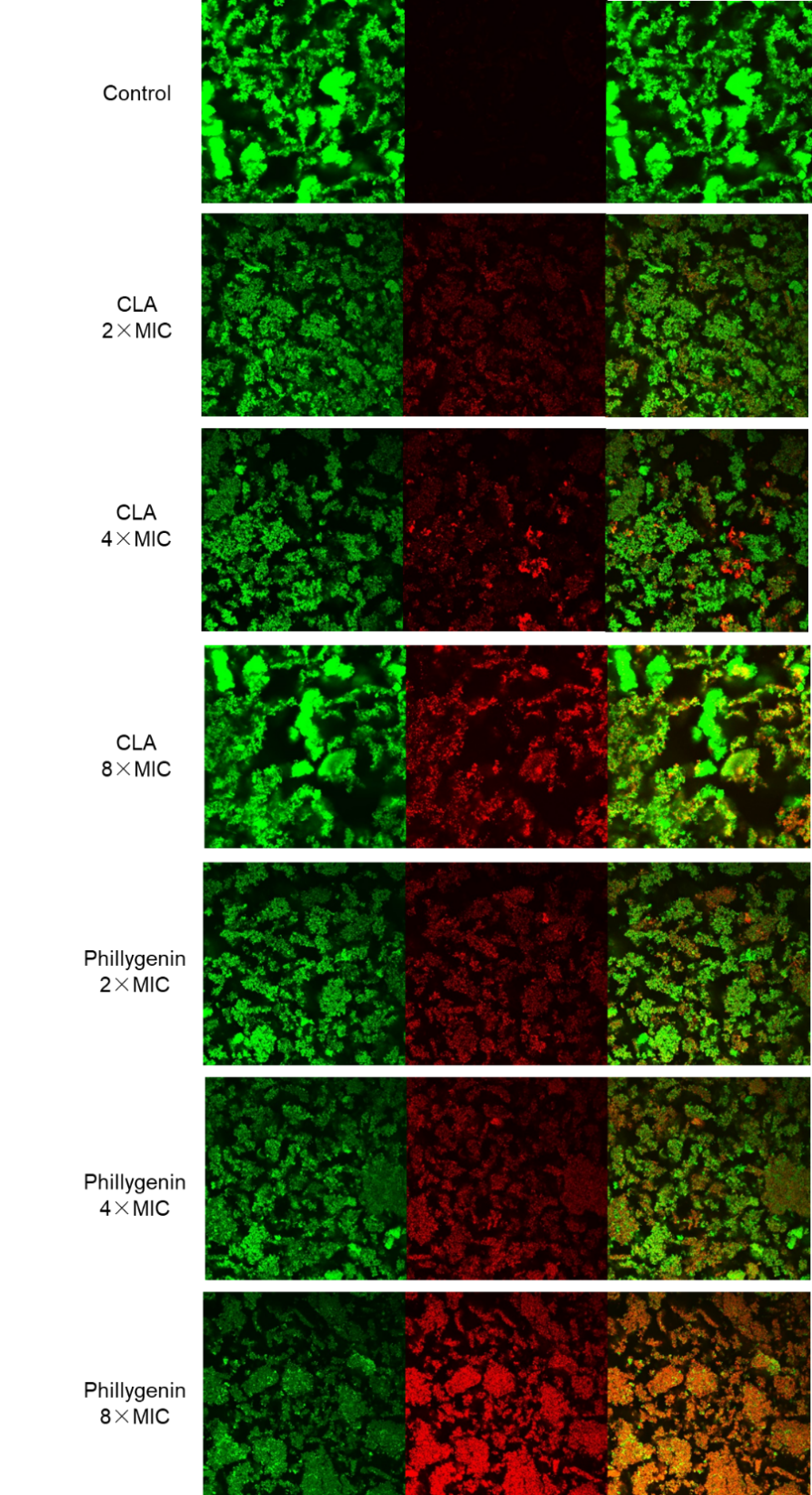


Figure 3 supplementary picture of the biofilm confocal experiment


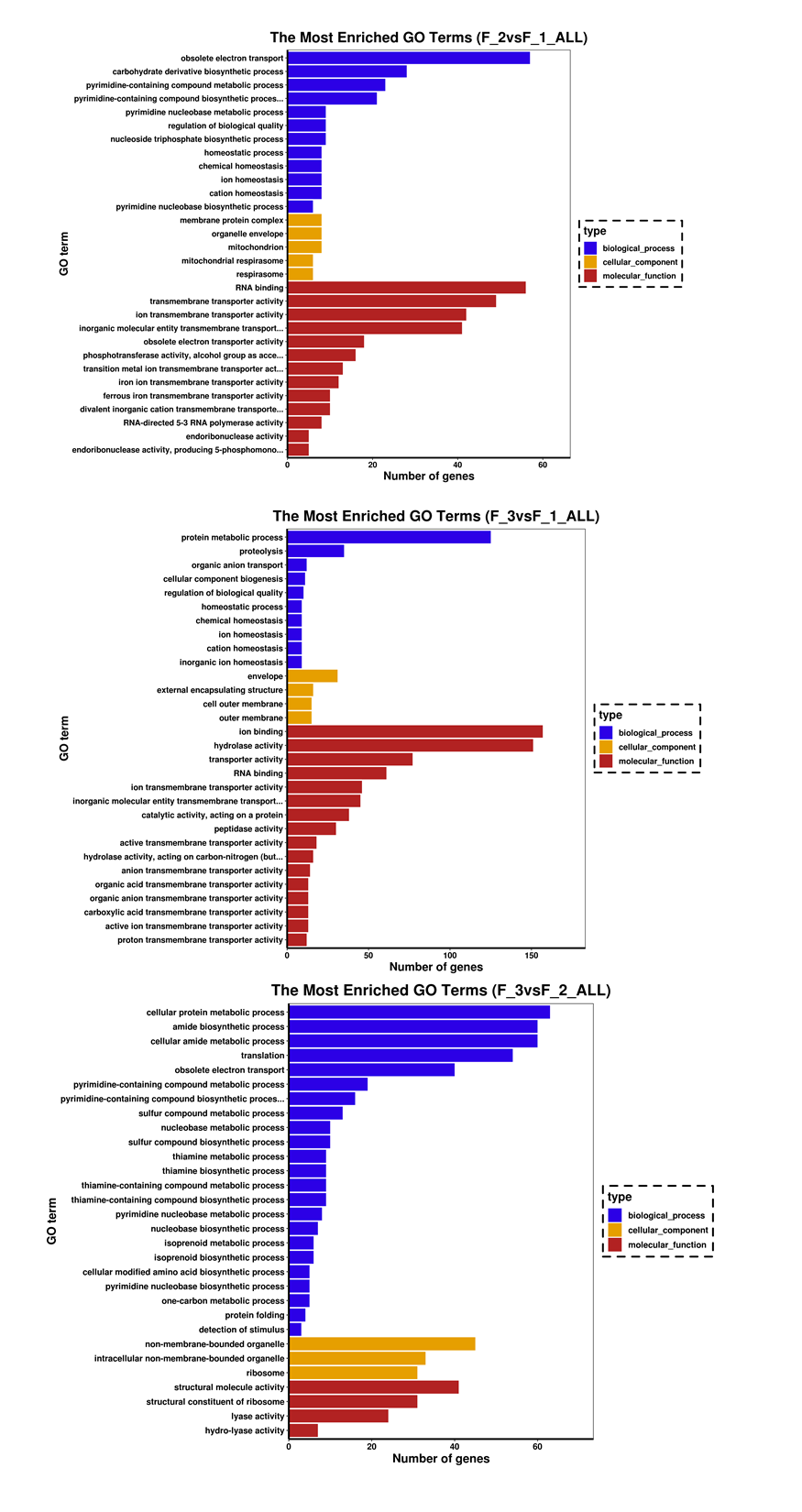


Figure 4 supplementary of GO enrichment histogram
